# Identification of Proteins Associated with an IFNγ-Responsive Promoter by a Retroviral Expression System for enChIP Using CRISPR

**DOI:** 10.1371/journal.pone.0103084

**Published:** 2014-07-22

**Authors:** Toshitsugu Fujita, Hodaka Fujii

**Affiliations:** Combined Program on Microbiology and Immunology, Research Institute for Microbial Diseases, Osaka University, Suita, Osaka, Japan; University of Nebraska - Lincoln, United States of America

## Abstract

Isolation of specific genomic regions retaining molecular interactions is essential for comprehensive identification of molecules associated with the genomic regions. Recently, we developed the engineered DNA-binding molecule-mediated chromatin immunoprecipitation (enChIP) technology for purification of specific genomic regions. Here, we developed a retroviral expression system for enChIP using CRISPR. We showed that the target genomic locus can be purified with high efficiency by using this system. We also showed that contamination of potential off-target sites is negligible by using this system if the guide RNA (gRNA) for the target site has a sufficiently long unique sequence in its seed sequence. enChIP combined with stable isotope labeling using amino acids in cell culture (SILAC) analysis identified proteins whose association with the *interferon (IFN) regulatory factor-1 (IRF-1)* promoter region increases in response to IFNγ stimulation. The list of the associated proteins contained many novel proteins in the context of IFNγ-induced gene expression as well as proteins related to histone deacetylase complexes whose involvement has been suggested in IFNγ-mediated gene expression. Finally, we confirmed IFNγ-induced increased association of the identified proteins with the *IRF-1* promoter by ChIP. Thus, our results showed that the retroviral enChIP system using CRISPR would be useful for biochemical analysis of genome functions including transcription and epigenetic regulation.

## Introduction

A comprehensive understanding of the mechanisms behind genome functions such as transcription and epigenetic regulation requires the identification of the molecules that bind to the genomic regions of interest *in vivo*. We previously developed the locus-specific chromatin immunoprecipitation (ChIP) technologies consisting of insertional ChIP (iChIP) [1]–[5] and engineered DNA-binding molecule-mediated ChIP (enChIP) [6], [7] for purification of specific genomic regions to identify their associated molecules. In enChIP, a tagged engineered DNA-binding molecule is expressed into the cell to be analyzed so that it recogizes an endogenous target DNA sequence. Subsequently, the target genomic region is subjected to affinity-purification such as immunoprecipitation with an antibody (Ab) against the tag(s). We showed that the clustered regularly interspaced short palindromic repeats (CRISPR) system [8]–[21] combined with enChIP efficiently isolates specific genomic regions for identification of their associated proteins [6]. In this form of enChIP, specific genomic regions are immunoprecipitated with an Ab against a tag(s), which is fused to a catalytically inactive form of Cas9 (dCas9) plus guide RNA (gRNA) interacting with an endogenous DNA sequence in the genomic regions (Figure S1). Because it is easy to generate gRNA targeting specific genomic regions, enChIP using the CRISPR system is a convenient way to perform enChIP analysis. In our previous paper, we used a transient transfection approach to express the CRISPR components [6]. The system would be applicable to those cell lines with high transfection efficiency. However, the transient transfection approach might not work for cells with low transfection efficiency. In those cases, stable expression of the CRISPR components would be necessary.

Here, we developed a retroviral expression system for enChIP using CRISPR. Stable isotope labeling using amino acids in cell culture (SILAC) [22] combined with enChIP (enChIP-SILAC) identified proteins induced to interact with the *interferon (IFN) regulatory factor-1 (IRF-1)* promoter region in response to IFNγ stimulation. The retroviral expression system for enChIP using CRISPR would be useful for biochemical analysis of genome functions such as transcription, epigenetic regulation, genomic imprinting, and X chromosome inactivation.

## Results

### Generation of a retroviral expression system for enChIP using CRISPR

To generate cells stably expressing the components of enChIP using CRISPR more easily and quickly, we developed a retroviral system to express 3xFLAG-dCas9 (dCas9 tagged with the 3xFLAG tag and fused with a nuclear localization signal (NLS)) [6] and gRNA. The coding sequence of 3xFLAG-dCas9 was inserted into pMXs [23]-derived retroviral expression vectors retaining various selection markers (Table 1). In addition, pSIR [24]–[26]-derived self-inactivating retroviral vectors with various selection markers were developed to express gRNA (Table 2). gBlock, an expression unit of gRNA, can be inserted into the multiple cloning sites of these vectors. To target the promoter region of human *IRF-1* gene [27], the gBlock of gRNA-hIRF-1 #12 [6] was inserted into pSIR to generate gRNA-hIRF-1 #12/pSIR.

**Table 1:** Retroviral vectors expressing 3xFLAG-dCas9.

*Plasmid*	*Selection marker*	*Addgene ID* #
3xFLAG-dCas9/pMXs-puro	Puromycin resistance gene	51240
3xFLAG-dCas9/pMXs-neo	Neomycin resistance gene	51260
3xFLAG-dCas9/pMXs-IG	GFP	51258
3xFLAG-dCas9/pMXs-I2	hCD2	51259

**Table 2:** Self-inactivating retroviral vectors.

*Plasmid*	*Selection marker*	*Examples of gBlock cloning sites*	*Addgene ID* #
pSIR-neo	Neomycin resistance gene	*Xho* I + *Hind* III	51128
pSIR-GFP	GFP	*Xho* I + *Hind* III, *EcoR* I	51134
pSIR-DsRed-Express2	DsRed-Express2	*Xho* I + *Hind* III, *EcoR* I	51135
pSIR-hCD2	hCD2	*EcoR* I	51143

**Figure 1:**
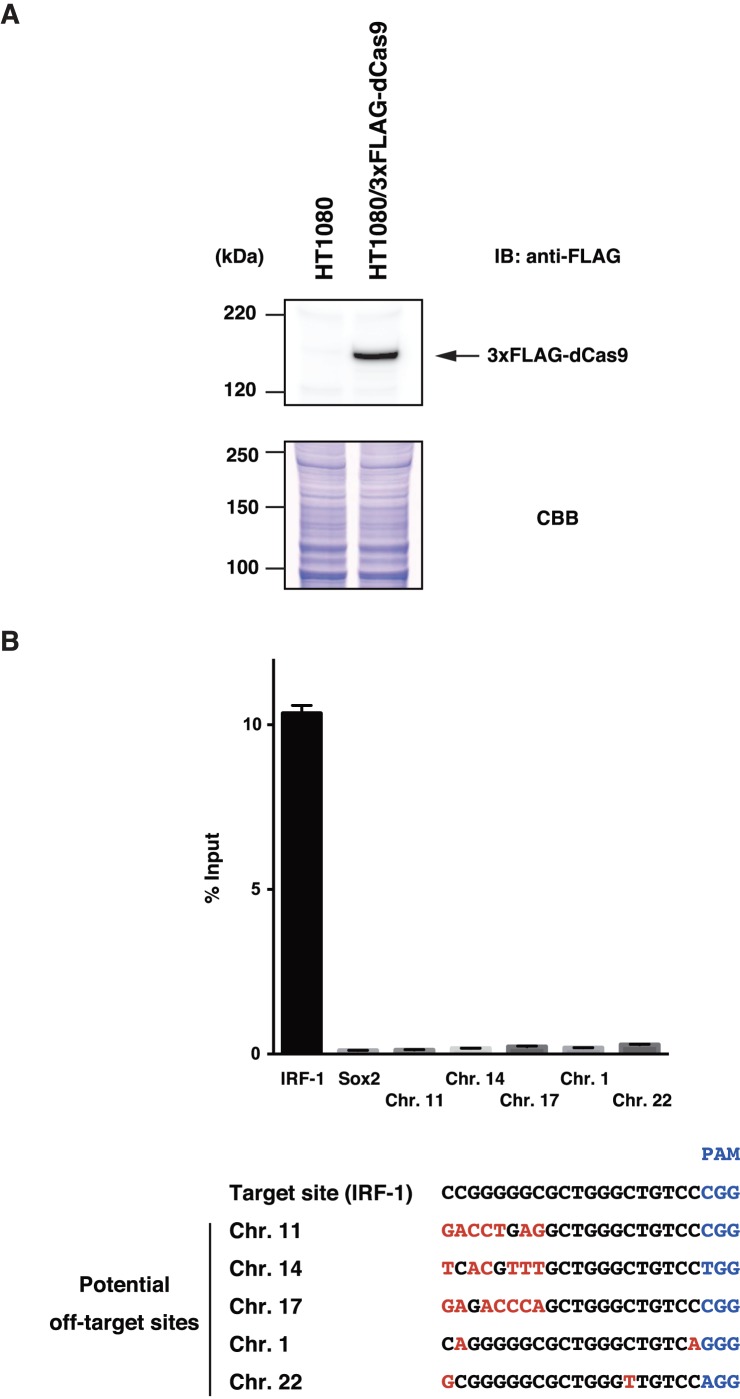
Yield of enChIP analysis for the target site and potential off-target sites. (**A**) Expression of 3xFLAG-dCas9 in HT1080-derived cells. Expression of 3xFLAG-dCas9 was detected by immunoblot analysis with anti-FLAG Ab. Coomassie Brilliant Blue (CBB) staining is shown as a protein loading control. (**B**) Upper panel: Yield of enChIP analysis for the target site and potential off-target sites (mean +/- SD, n = 3). Lower panel: Alignment of the target site and potential off-target sites. The PAM sequences and mismatches are shown in blue and red, respectively.

To examine if the system works, 3xFLAG-dCas9/pMXs-puro was transduced into a human fibrosarcoma cell line, HT1080. After puromycin selection, expression of 3xFLAG-dCas9 was confirmed by immunoblot analysis with anti-FLAG Ab (Figure 1A) (the full-length images with size markers are shown in Figure S2). Subsequently, gRNA-hIRF-1 #12/pSIR was transduced into the HT1080 cells expressing 3xFLAG-dCas9. Cells expressing the gRNA were selected with G418.

### Yield of enChIP for the target site and potential off-target sites

Next, we examined yield of enChIP for the target *IRF-1* promoter locus. The cells expressing 3xFLAG-dCas9 and gRNA-hIRF-1 #12 were crosslinked with formaldehyde, and crosslinked chromatin was fragmented by sonication. Complexes containing 3xFLAG-dCas9 and gRNA-hIRF-1 #12 were immunoprecipitated with anti-FLAG Ab. Real-time PCR showed that around 10% of input genomic DNA was immunoprecipitated for the target *IRF-1* promoter locus (Figure 1B). This yield was comparable with that observed in 293T cells transiently transfected with plasmids expressing 3xFLAG-dCas9 and gRNA-hIRF-1 #12 [6]. We also tested the retroviral enChIP system using CRISPR for a human leukemia cell line, K562. The *IRF-1* promoter region was also specifically isolated from K562-derived cells (Figure S3). These results indicated that efficient purification of target genomic regions is feasible by using the retroviral expression system for enChIP using CRISPR.

Next, we examined yield for potential off-target sites. CRISPR tolerates mismatches in the 5′ region of target sites but not in the Protospacer Adjacent Motif (PAM) sequence and the seed sequence 5′ proximal to PAM [10]. No other site in the human genome contains sequences identical to 16-base of the seed sequence of gRNA-hIRF-1 #12 including the PAM sequence. Sites in chromosomes 11, 14, and 17 have the identical 15-base sequences in their seed sequences including PAM as well as mismatches in the 5′ side of the identical 15-base sequences (Figure 1B). Sites in chromosomes 1 and 22, which are the most similar to the target site in the human genome, have two-base mismatches, one of which is present in the seed sequences near PAM. As shown in Figure 1B, yield for the potential off-target sites was marginal and comparable to that for the irrelevant *Sox2* locus, suggesting that contamination of potential off-target sites can be minimal when target sites have more than a 16-base long unique sequence in the seed sequence including PAM.

### Induction of expression of the *IRF-1* gene in the presence of 3xFLAG-dCas9 and gRNA

**Figure 2:**
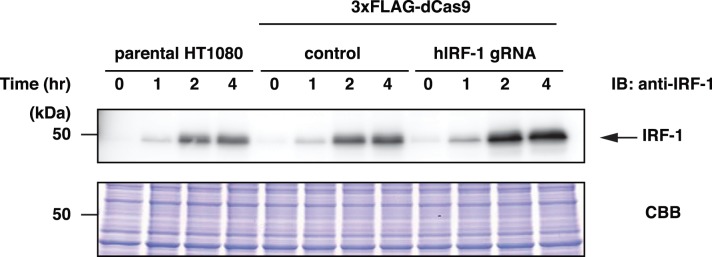
IFNγ-induced expression of IRF-1. HT1080 and its derived cells were stimulated with 100 ng/ml of IFNγ for indicated time intervals. Nuclear extracts were subjected to SDS-PAGE and immunoblot analysis with anti-IRF-1 Ab. CBB staining is shown as a protein loading control.

Binding of the CRISPR complexes may interfere with gene expression (CRISPRi) [21]. To examine whether *IRF-1* gene expression might be abrogated by the binding of 3xFLAG-dCas9 and the gRNA targeting the *IRF-1* promoter, we performed immunoblot analysis of the IRF-1 protein after stimulation with IFNγ in cells derived from HT1080, in which IFNγ stimulation is known to induce IRF-1 expression [28]. As shown in Figure 2 (the full-length images with size markers are shown in Figure S4), IFNγ induced expression of IRF-1 even in the presence of 3xFLAG-dCas9 and gRNA targeting the *IRF-1* promoter. This result suggested that binding of 3xFLAG-dCas9 and gRNA-hIRF-1 #12 does not abolish *IRF-1* transcription.

### enChIP-SILAC analysis to quantitatively detect changes in the amounts of proteins associated with the *IRF-1* promoter in response to IFNγ stimulation

Next, we performed enChIP-SILAC of the HT1080-derived cells expressing 3xFLAG-dCas9 and gRNA-hIRF-1 #12 to identify proteins whose interaction with the *IRF-1* promoter changes in response to IFNγ stimulation (Figure S5). In SILAC, cells are differentially labeled by culturing them in Light medium containing normal amino acids or Heavy medium with amino acids such as Lysine and Arginine conatining heavy isotopes. The mass shift of proteins resulted by metabolic incorporation of the Light and Heavy amino acids is detected by mass spectrometry. For quantification of detected proteins, Heavy to Light protein ratios (Heavy/Light values) are calculated within samples by summing average intensity values for all heavy peptides for each protein and dividing by the corresponding light values [22]. In our experimental settings, the Heavy/Light values more than 1 indicated that the identified proteins were detected more abundantly from cells stimulated for 30 min with IFNγ than from the mock-stimulated cells. We detected a list of proteins whose interactions with the *IRF-1* promoter change by IFNγ stimulation (Table 3 and Table S1).

**Table 3:** Examples of proteins identified by enChIP-SILAC.

Categories	Proteins
Transcription	DDX1, PARP1, CKAP4, Pescadillo homolog, PURβ, activated RNA polymerase II transcriptional activator p15, BTF3, Myb-binding protein 1A
Histone deacetylation, corepressor components	RBBP4, PA2G4, TBL3
Acetyltransferase	Protein arginine N-methyltransferase 1
DNA topoisomerase	DNA topoisomerase 2α
Histones	Histone H2A.Z, histone H3.2

All of identified proteins, identified peptides, and the raw Heavy/Light value are shown in Table S1.

In the list of proteins whose association with the *IRF-1* promoter increased by IFNγ stimulation, we detected several classes of proteins: (i) proteins whose involvement in transcriptional regulation is suggested including DDX1 [29], PARP1 [30]–[32], CKAP4 [33], Pescadillo homolog [34], transcriptional activator protein PURβ [35], activated RNA polymerase II transcriptional activator p15 (TCP4) [36], [37], BTF3 [38], and Myb-binding protein 1A [39], (ii) proteins involved in histone deacetylation and/or corepressor function including RBBP4 [40], [41], PA2G4 [42], and TBL3 [43], [44], (iii) protein arginine N-methyltransferase 1 (PRMT1) [45], [46], (iv) DNA topoisomerase 2α [47], and (v) histones including histone H2A.Z and histone H3.2.

### IFNγ-induced increased association of the candidate proteins with the *IRF-1* promoter

**Figure 3:**
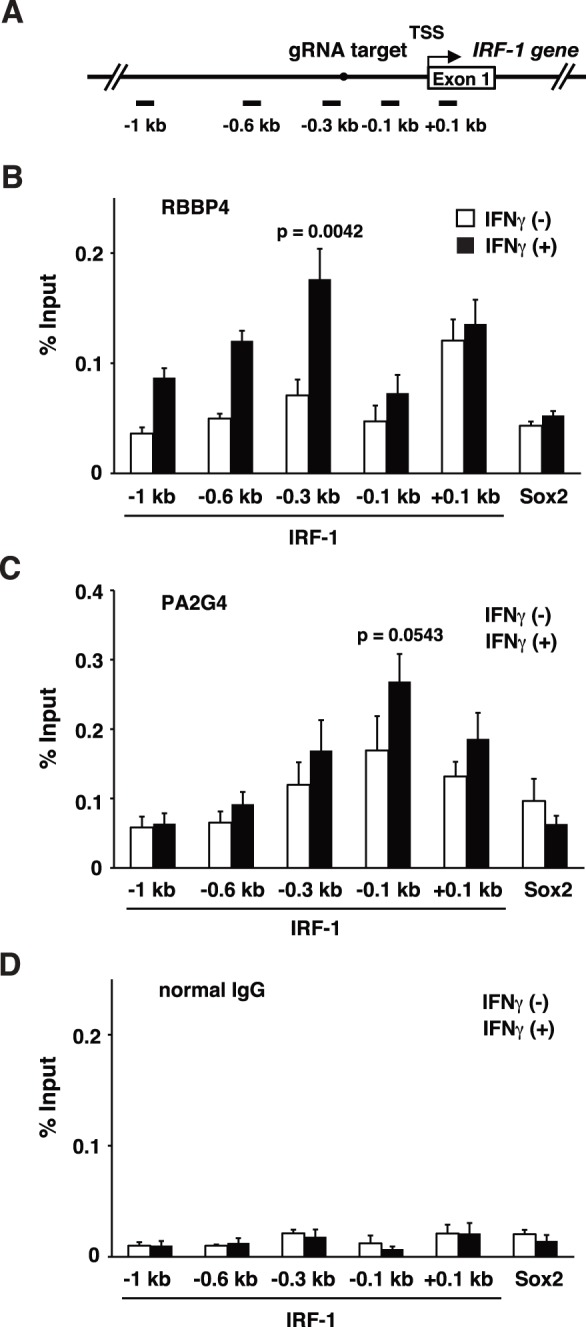
IFNγ-induced increased association of candidate proteins with the *IRF-1* promoter. (**A**) A scheme of human *IRF-1* promoter. TSS: transcription start site; gRNA target: the position of the target sequence of gRNA. The positions of PCR primers for ChIP with distances from TSS are indicated. The results of ChIP using (**B**) anti-RBBP4 Ab, (**C**) anti-PA2G4 Ab, and (**D**) negative control normal IgG, are shown (mean +/- SD, n = 3). IFNγ-induced increase in binding of RBBP4 and PA2G4 to the *IRF-1* promoter was reproducibly detected by ChIP analysis.

To confirm increased association of candidate proteins with the *IRF-1* promoter by IFNγ stimulation, we attempted to perform ChIP analysis using Abs against endogenous proteins. In this regard, involvement of histone deacetylation has been implicated in IFNγ-induced gene expression [48], [49]. However, molecules involved in IFNγ-induced histone deacetylation were not fully revealed. Therefore, we chose proteins involved in histone deacetylation and/or corepressor function for ChIP analysis. As shown in Figure 3, IFNγ stimulation increased binding of RBBP4 and PA2G4 to the *IRF-1* promoter, whereas their binding to the control *Sox2* locus showed a marginal change. IFNγ induced increase in binding of RBBP4 at the distal region of the *IRF-1* promoter (between −1 kb and −0.3 kb from the transcription start site (TSS)) (Figure 3B), whereas association of PA2G4 increased in the proximal region (between −0.3 kb and +0.1 kb from TSS) by IFNγ stimulation (Figure 3C). In this experimental setting, we confirmed IFNγ-induced association of Stat1 with the *IRF-1* promoter (Figure S6) to ensure validity of stimulation conditions. This result clearly showed that enChIP-SILAC is able to detect relevant proteins whose association with the target genomic regions changes in response to extracellular stimuli.

## Discussion

In this study, we developed a retroviral enChIP system using CRISPR. In our previous paper of enChIP using CRISPR, we used a transient transfection approach [6]. The approach worked well in those cell lines with high transfection efficiency such as 293T cells. However, the transient transfection approach might be difficult to use for cells with low transfection efficiency. In those cases, stable expression of components of the CRISPR system would be necessary. Retroviral transduction of enChIP components enables us to establish stable cell lines in a shorter period of time and with less effort than conventional transfection strategies. We showed that the retroviral enChIP system is able to purify the targeted genomic regions efficiently and specifically from cell lines including HT1080 (Figure 1) and K562 (Figure S3). Other endogenous loci were also specifically purified from K562-derived cells by enChIP (T.F. and H.F., unpublished data). The yield of the retroviral enChIP system using HT1080-derived cells was comparable with that observed in 293T cells transiently transfected with 3xFLAG-dCas9 and gRNA [6].

It has been suggested that one of potential drawbacks of the CRISPR system is off-target effects [50]–[53]. Therefore, we examined if contamination of potential off-target sites has a big impact on the results of enChIP using CRISPR. As shown in Figure 1B, yield for potential off-target sites was negligible compared with that of the target site when the gRNAs for target sites have more than 16-base long unique sequences in their seed sequences. It would be likely that contamination of potential off-target sites would become larger as the target site has a shorter unique sequence. Our results also indicated importance of the seed sequence near PAM because a single base mismatch near PAM significantly decreased the yield (potential off-target sites in chromosomes 1 and 22). These results are consistent with the data showing that cleavage of target DNA by wild-type Cas9 is governed by the seed sequence of gRNA [10], [15] and a recent report that DNA strand separation and RNA-DNA heteroduplex formation initiate at PAM and proceed directionally towards the distal end of the target sequence [54]. Our data might be informative to design gRNA for enChIP using CRISPR.

Next, by using the retroviral enChIP system, we attempted to identify proteins associated with the *IRF-1* promoter in an IFNγ-dependent manner. We detected a list of proteins whose association increases upon IFNγ stimulation (Table 3 and Table S1). The list contained proteins involved in transcriptional regulation and other genome functions. Especially, the fact that the enChIP-SILAC analysis identified proteins involved in histone deacetylation and/or corepressor function is consistent with the previous reports that histone deacetylases positively regulate IFNγ-induced gene expression [48], [49]. For example, HDAC1 has been implicated in IFNγ-induced gene expression [48].

Finally, we confirmed IFNγ-induced association of two of the identified proteins, RBBP4 and PA2G4, with the *IRF-1* promoter by ChIP (Figure 3). It has been shown that RBBP4 and PA2G4 are involved in histone deacetylation and/or corepressor function [40]–[42], but their involvement in IFNγ-induced gene expression has not been documented. Considering that we also detected TBL3 [43], [44], another component of histone deacetylase/corepressor complexes, in enChIP-SILAC analysis, it is likely that the histone deacetylase/corepressor complexes containing these proteins play important roles in IFNγ-induced transcription together with HDACs such as HDAC1. Elucidation of the functions of the proteins identified in this study in IFNγ-induced gene expression would be an interesting future issue. Since we detected many proteins in our enChIP-SILAC analysis, it would be a challenge to make a comprehensive view on their roles in IFNγ-induced gene expression at this stage. Systems biology approaches in the analysis of the detected proteins would be an attractive option in the future analysis.

## Conclusions

In this study, we developed a retroviral enChIP system using CRISPR. We showed that the target genomic locus could be purified with high efficiency by using this system (Figure 1). We also showed that contamination of potential off-target sites is negligible by using this system if gRNAs for the target sites have sufficiently long unique sequences in their seed sequences (Figure 1). enChIP-SILAC analysis identified proteins induced to bind to the *IRF-1* promoter region in response to IFNγ stimulation (Table 3). The list of the associated proteins contained many novel proteins in the context of IFNγ-induced gene expression as well as proteins related to histone deacetylase complexes whose involvement has been suggested in IFNγ-induced gene expression (Table 3). We confirmed IFNγ-induced association of identified proteins with the *IRF-1* promoter (Figure 3). Thus, our results showed that the retroviral enChIP system using CRISPR would be useful for biochemical analysis of genome functions.

## Materials and Methods

### Plasmid construction

To construct 3xFLAG-dCas9/pMXs-puro, 3xFLAG-dCas9/pCMV-7.1 [6] was digested with *Sac* I. The 1.7 kbp fragment containing 3xFLAG and the N-terminal portion of dCas9 was blunted and inserted into the pMXs-puro vector [7], which was digested with *Bam*H I and blunted, to generate 3xFLAG-dCas9-N/pMXs-puro. Subsequently, 3xFLAG-dCas9/pCMV-7.1 was digested with *Kpn* I, blunted, and cleaved with *Sbf* I, and the 3.9 kbp fragment containing the C-terminal portion of dCas9 plus NLS was inserted into the 3xFLAG-dCas9-N/pMXs-puro plasmid, which was digested with *Eco*R I, blunted, and cleaved with *Sbf* I to generate 3xFLAG-dCas9/pMXs-puro.

To generate 3xFLAG-dCas9/pMXs-IG, 3xFLAG-dCas9/pMXs-I2, and 3xFLAG-dCas9/pMXs-neo, the coding sequence of 3xFLAG-dCas9, which was isolated from 3xFLAG-dCas9/pMXs-puro by digestion with *Pac* I and *Not* I, was inserted into pMXs-IG [23], pMXs-I2 [3], and pMXs-neo [7], respectively.

To construct gRNA-hIRF-1 #12/pSIR, the gBlock isolated from gRNA-hIRF-1 #12 [6] by *Xho* I and *Hin*d III digestion was inserted into the *Xho* I- and *Hin*d III-digested pSIR self-inactivating retrovirus vector (Clontech).

To construct pSIR-hCD2, pSIR-GFP, and pSIR-DsRed-Express2, the neomycin-resistance gene of pSIR was replaced with human CD2, GFP, or DsRed-Express2 gene, respectively. To construct pSIR-neo, the multicloning site of pMX [7] was inserted into *Xho* I- and *Hin*d III-cleaved pSIR.

The plasmids are available through Addgene: 3xFLAG-dCas9/pMXs-puro (51240), 3xFLAG-dCas9/pMXs-IG (51258), 3xFLAG-dCas9/pMXs-I2 (51259), 3xFLAG-dCas9/pMXs-neo (51260), pSIR-neo (51128), pSIR-GFP (51134), pSIR-DsRed-Express2 (51135), and pSIR-hCD2 (51143).

### Cell lines

The HT1080 cell line [55] was purchased from ATCC (CCL-121). HT1080-derived cells were maintained in DMEM (Wako) supplemented with 10% fetal calf serum (FCS). The K562 cell line [56] was obtained from RIKEN BioResource Center (RCB0027). K562-derived cells were maintained in RPMI (Wako) supplemented with 10% FCS.

### Establishment of cells stably expressing 3xFLAG-dCas9 and gRNA

For establishment of cells expressing 3xFLAG-dCas9, 5 µg of 3xFLAG-dCas9/pMXs-puro together with 5 µg of an amphotropic helper plasmid, pPAM3 [57], was transfected into 1×10^6^ of 293T cells using Lipofectamine 2000 (Life Technologies) according to the instructions by the manufacturer to produce retrovirus particles. Two days after transfection, HT1080 cells or K562 cells were infected with the supernatant (5 ml) of the 293T cells containing the virus particles. HT1080- or K562-derived cells expressing 3xFLAG-dCas9 were selected in DMEM or RPMI medium containing 10% FCS and puromycin (0.5 µg/ml), respectively.

For establishment of cells expressing both 3xFLAG-dCas9 and the gRNA targeting the *IRF-1* locus, 5 µg (for HT1080-derived cells) or 2 µg (for K562-derived cells) of gRNA-hIRF-1 #12/pSIR was transfected into 1×10^6^ of 293T cells together with 5 µg (for HT1080-derived cells) or 2 µg (for K562-derived cells) of pPAM3. Two days after transfection, the HT1080- or K562-derived cells expressing 3xFLAG-dCas9 were infected with the supernatant (5 ml) of the 293T cells containing the virus particles. HT1080- or K562-derived cells expressing both 3xFLAG-dCas9 and gRNA-hIRF-1 #12 were selected in DMEM or RPMI medium containing 10% FCS, puromycin (0.5 µg/ml), and G418 (0.8 mg/ml).

### Immunoblot analysis

Expression of 3xFLAG-dCas9 was detected by immunoblot analysis with anti-FLAG M2 Ab (F1804, Sigma-Aldrich) as described previously [6]. For detection of the IRF-1 protein, HT1080 and its derived cells were stimulated with 100 ng/ml of recombinant human IFNγ (rhIFNγ). Nuclear extracts (NE) were prepared with NE-PER Nuclear and Cytoplasmic Extraction Reagents (Thermo Fisher Scientific). 7.5 µg of NE were subjected to immunoblot analysis with anti-human IRF-1 Ab (C-20, Santa Cruz Biotechnology).

### Enchip-real-time PCR

enChIP-real-time PCR was performed as previously described [6]. Primers used in the analysis are shown in Table S2.

### Enchip-SILAC

The HT1080 cells expressing 3xFLAG-dCas9 and gRNA-hIRF-1 #12 were grown in DMEM and FCS provided in Pierce SILAC Protein Quantitation Kit - DMEM (Thermo Fisher Scientific) with Lysine-2HCl and L-Arginine-HCl (Thermo Fisher Scientific) (Light medium) or ^13^C_6 _L-Lysine-2HCl and ^13^C_6_^15^N_4_L_–_Arginine-HCl (Thermo Fisher Scientific) (Heavy medium) according to the manufacture’s instructions. 5×10^7^ of isotopically labeled cells were stimulated with rhIFNγ (100 ng/ml) for 30 min and mixed with 5×10^7^ of control cells cultured in Light medium. Cells were fixed with 1% formaldehyde at 37°C for 5 min. The chromatin fraction was extracted and fragmented by sonication (the average length of fragments was about 2 kbp) as described previously [58] except for using 4 ml of Sonication Buffer (10 mM Tris-HCl (pH 8.0), 150 mM NaCl, 1 mM EDTA, 0.5 mM EGTA, 0.1% sodium deoxycholate, 0.1% SDS, complete protease inhibitor cocktail without EDTA (Roche)) and Ultrasonic disruptor UD-201 (Tomy Seiko). The sonicated chromatin in Sonication Buffer with 1% TritonX-100 was pre-cleared with 75 µg of normal mouse IgG (Santa Cruz Biotechnology) conjugated to 750 µl of Dynabeads-Protein G (Invitrogen) and subsequently incubated with 75 µg of anti-FLAG M2 Ab conjugated to 750 µl of Dynabeads-Protein G at 4°C for 20 h. The Dynabeads were washed twice each with 1.5 ml of Low Salt Wash Buffer (20 mM Tris-HCl (pH 8.0), 150 mM NaCl, 2 mM EDTA, 1% TritonX-100, 0.1% SDS, complete protease inhibitor cocktail without EDTA), High Salt Wash Buffer (20 mM Tris-HCl (pH 8.0), 500 mM NaCl, 2 mM EDTA, 1% TritonX-100, 0.1% SDS, complete protease inhibitor cocktail without EDTA), and LiCl Wash Buffer (10 mM Tris-HCl (pH 8.0), 250 mM LiCl, 1 mM EDTA, 0.5% IGEPAL-CA630, 0.5% sodium deoxycholate, complete protease inhibitor cocktail without EDTA), and once with 1.5 ml of TBS Buffer (50 mM Tris-HCl (pH 7.5), 150 mM NaCl) with 0.1% IGEPAL-CA630 and complete protease inhibitor cocktail without EDTA. The immunoprecipitants were eluted with 400 µl of Elution Buffer (500 µg/ml 3xFLAG peptide (Sigma-Aldrich), 50 mM Tris-HCl (pH 7.5), 150 mM NaCl, 0.1% IGEPAL-CA630, complete protease inhibitor cocktail without EDTA) at 37°C for 20 min. The eluted chromatin complexes were precipitated by adding 1 ml of 2-propanol with 50 µl of 3M sodium acetate and 5 µl of 20 mg/ml glycogen at −20°C overnight. After centrifugation (17,400× g) at 4°C for 30 min, the precipitants were washed with 1 ml of 70% ethanol and then incubated in 50 µl of 2× Sample Buffer (125 mM Tris-HCl (pH 6.8), 10% 2-mercaptoethanol, 4% SDS, 10% sucrose, 0.004% bromophenol blue) at 98°C for 30 min for reverse-crosslinking and denaturation of proteins. The reverse-crosslinked proteins were subjected to SDS-PAGE and visualized by staining with Coomassie Brilliant Blue (Bio-Rad). Visualized proteins were excised and analyzed using a nanoLC-MS/MS system composed of LTQ Orbitrap Velos (Thermo Fisher Scientific) coupled with nanoLC (Advance, Michrom BioResources) and HTC-PAL autosampler (CTC Analytics) at DNA-chip Development Center for Infectious Diseases (RIMD, Osaka University).

### ChIP

HT1080 cells were stimulated with 100 ng/ml of rhIFNγ at 37°C for 30 min. The cells (2×10^7^) were fixed with 1% formaldehyde at 37°C for 5 min. The chromatin fraction was extracted and fragmented by sonication as described previously [58] except for using 800 µl of of Sonication Buffer and Ultrasonic disruptor UD-201. 160 µl of the sonicated chromatin in Sonication Buffer with 1% Triton X-100 was pre-cleared with 5 µg of normal rabbit IgG conjugated to 30 µl of Dynabeads-Protein G and subsequently incubated with 5 µg of anti-PA2G4/EBP1 Ab (ab33613, Abcam), anti-RBBP4/RbAp48 Ab (ab38135, Abcam), or normal rabbit IgG conjugated to 30 µl of Dynabeads-Protein G at 4°C for 20 h. The immunoprecipitants were washed once each with 1 ml of Low Salt Wash Buffer, High Salt Wash Buffer, LiCl Wash Buffer, and TE Buffer (10 mM Tris-HCl (pH 8.0), 1 mM EDTA). After reverse-crosslinking at 65°C for at least 4 h, the DNA was purified with ChIP DNA Clean & Concentrator (Zymo Research) and used as template for real-time PCR with SYBR Select PCR system (Applied Biosystems) using the Applied Biosystems 7900HT Fast Real-Time PCR System. PCR cycles were as follows: heating at 50°C for 2 min followed by 95°C for 10 min; 40 cycles of 95°C for 15 sec and 60°C for 1 min. The primers used in this experiment are shown in Table S2.

### Statistical analysis

p-values were calculated with the Prism software (Graphpad) usint *t* test.

## Supporting Information

Figure S1 The scheme of enChIP using CRISPR.
(PDF)

Figure S2 The full-length images of Figure 1A including molecular size markers.
(PDF)

Figure S3 Specific isolation of the *IRF-1* promoter region from K562-derived cells by enChIP using CRISPR.
(PDF)

Figure S4 The full-length images of Figure 2 including molecular size markers.
(PDF)

Figure S5 The scheme of enChIP-SILAC.
(PDF)

Figure S6 IFNγ-induced association of Stat1 with the *IRF-1* promoter.
(PDF)

Table S1 List of proteins detected in enChIP-SILAC.
(XLSX)

Table S2 Primers used in this study.
(PDF)

## References

[1] Hoshino A, Fujii H (2009) Insertional chromatin immunoprecipitation: a method for isolating specific genomic regions. J Biosci Bioeng 108: 446–449. 19804873 10.1016/j.jbiosc.2009.05.005

[2] Fujita T, Fujii H (2011) Direct idenification of insulator components by insertional chromatin immunoprecipitation. PLoS One 6: e26109. 22043306 10.1371/journal.pone.0026109 PMC3197142

[3] Fujita T, Fujii H (2012) Efficient isolation of specific genomic regions by insertional chromatin immunoprecipitation (iChIP) with a second-generation tagged LexA DNA-binding domain. Adv Biosci Biotechnol 3: 626–629.

[4] Fujita T, Fujii H (2013) Locus-specific biochemical epigenetics/chromatin biochemistry by insertional chromatin immunoprecipitation. ISRN Biochem 2013: Article ID 913273. 10.1155/2013/913273 PMC4392943 25969763

[5] Fujita T, Fujii H (2014) Efficient isolation of specific genomic regions retaining molecular interactions by the iChIP system using recombinant exogenous DNA-binding proteins. bioRxiv: http://dx.doi.org/doi:10.1101/006080. 10.1186/s12867-014-0026-0 PMC4253623 25428274

[6] Fujita T, Fujii H (2013) Efficient isolation of specific genomic regions and identification of associated proteins by engineered DNA-binding molecule-mediated chromatin immunoprecipitation (enChIP) using CRISPR. Biochem Biophys Res Commun 439: 132–136. 23942116 10.1016/j.bbrc.2013.08.013

[7] Fujita T, Asano Y, Ohtsuka J, Takada Y, Saito K, et al (2013) Identification of telomere-associated molecules by engineered DNA-binding molecule-mediated chromatin immunoprecipitation (enChIP). Sci Rep 3: 3171. 24201379 10.1038/srep03171 PMC3821016

[8] Makarova KS, Haft DH, Barrangou R, Brouns SJ, Charpentier E, et al (2011) Evolution and classification of the CRISPR-Cas systems. Nat Rev Microbiol 9: 467–477. 21552286 10.1038/nrmicro2577 PMC3380444

[9] Wiedenheft B, Sternberg SH, Doudna JA (2012) RNA-guided genetic silencing systems in bacteria and archaea. Nature 482: 331–338. 22337052 10.1038/nature10886

[10] Jinek M, Chylinski K, Fonfara I, Hauer M, Doudna JA, et al (2012) A programmable dual-RNA-guided DNA endonuclease in adaptive bacterial immunity. Science 337: 816–820. 22745249 10.1126/science.1225829 PMC6286148

[11] Gasiunas G, Barrangou R, Horvath P, Siksnys V (2012) Cas9-crRNA ribonucleoprotein complex mediates specific DNA cleavage for adaptive immunity in bacteria. Proc Natl Acad Sci USA 109: E2579–E2586. 22949671 10.1073/pnas.1208507109 PMC3465414

[12] Deltcheva E, Chylinski K, Sharma CM, Gonzales K, Chao Y, et al (2011) CRISPR RNA maturation by trans-encoded small RNA and host factor RNase III. Nature 471: 602–607. 21455174 10.1038/nature09886 PMC3070239

[13] Marraffini LA, Sontheimer EJ (2010) CRISPR interference: RNA-directed adaptive immunity in bacteria and archaea. Nat Rev Genet 11: 181–190. 20125085 10.1038/nrg2749 PMC2928866

[14] Mali P, Yang L, Esvelt KM, Aach J, Guell M, et al (2013) RNA-guided human genome engineering via Cas9. Science 339: 823–826. 23287722 10.1126/science.1232033 PMC3712628

[15] Cong L, Ran FA, Cox D, Lin S, Barretto R, et al (2013) Multiplex genome engineering using CRISPR/Cas system. Science 339: 819–823. 23287718 10.1126/science.1231143 PMC3795411

[16] Jinek M, East A, Cheng A, Lin S, Ma E, et al (2013) RNA-programmed genome editing in human cells. eLife 2: e00471. 23386978 10.7554/eLife.00471 PMC3557905

[17] Jiang W, Bikard D, Cox D, Zhang F, Marraffini LA (2013) RNA-guided editing of bacterial genomes using CRISPR-Cas systems. Nat Biotechnol 31: 233–239. 23360965 10.1038/nbt.2508 PMC3748948

[18] Hwang WY, Fu Y, Reyon D, Maeder ML, Tsai SQ, et al (2013) Efficient genome editing in zebrafish using a CRISPR-Cas system. Nat Biotechnol 31: 227–229. 23360964 10.1038/nbt.2501 PMC3686313

[19] Cho SW, Kim S, Kim JM, Kim J-S (2013) Targeted genome engineering in human cells with the Cas9 RNA-guided endonuclease. Nat Biotechnol 31: 230–232. 23360966 10.1038/nbt.2507

[20] Wang H-G, Yang H, Shivalila CS, Dawlaty MM, Cheng AW, et al (2013) One-step generation of mice carrying mutations in multiple genes by CRISPR/Cas-mediated genome engineering. Cell 153: 910–918. 23643243 10.1016/j.cell.2013.04.025 PMC3969854

[21] Qi LS, Larson MH, Gilbert LA, Doudna JA, Weissman JS, et al (2013) Repurposing CRISPR as an RNA-guided platform for sequence-specific control of gene expression. Cell 152: 1173–1183. 23452860 10.1016/j.cell.2013.02.022 PMC3664290

[22] Ong SE, Biagoev B, Kratchmarova I, Kristensen DB, Steen H, et al (2002) Stable isotope labeling by amino acids in cell culture, SILAC, as a simple and accurate approach to expression proteomics. Mol Cell Proteomics 1: 376–386. 12118079 10.1074/mcp.m200025-mcp200

[23] Nosaka T, Kawashima T, Misawa K, Ikuta K, Mui AL, et al (1999) STAT5 as a molecular regulator of proliferation, differentiation and apoptosis in hematopoietic cells. EMBO Journal 18: 4754–4765. 10469654 10.1093/emboj/18.17.4754 PMC1171548

[24] Yu S, von Rüden T, Kantoff PW, Garber C, Seiberg M, et al (1986) Self-inactivating retroviral vectors designed for transfer of whole genes into mammalian cells. Proc Natl Acad Sci U S A 83: 3194–3198. 3458176 10.1073/pnas.83.10.3194 PMC323479

[25] Nakajima K, Ikenaka K, Nakahira K, Morita N, Mikoshiba K (1993) An improved retroviral vector for assaying promoter activity. Analysis of promoter interference in pIP211 vector. FEBS Lett 315: 129–133. 8417968 10.1016/0014-5793(93)81148-s

[26] Guild BC, Finer MH, Housman DE, Mulligan RC (1988) Development of retrovirus vectors useful for expressing genes in cultured murine embryonal cells and hematopoietic cells in vivo. J Virol 62: 3795–3801. 3418785 10.1128/jvi.62.10.3795-3801.1988 PMC253524

[27] Miyamoto M, Fujita T, Kimura Y, Maruyama M, Harada H, et al (1988) Regulated expression of a gene encoding a nuclear factor, IRF-1, that specifically binds to IFN-beta gene regulatory elements. Cell 54: 903–913. 3409321 10.1016/s0092-8674(88)91307-4

[28] Hoshino A, Saint Fleur S, Fujii H (2006) Regulation of Stat1 protein expression by phenylalanine 172 in the coiled-coil domain. Biochem Biophys Res Commun 346: 1062–1066. 16782051 10.1016/j.bbrc.2006.06.026 PMC1861812

[29] Ishaq M, Ma L, Wu X, Mu Y, Pan J, et al (2009) The DEAD-box RNA helicase DDX1 interacts with RelA and enhances nuclear factor kappaB-mediated transcription. J Cell Biochem 106: 296–305. 19058135 10.1002/jcb.22004

[30] Aprile-Garcia F, Antunica-Noguerol M, Budziñski ML, Liberman AC, Arzt E (2013) Novel insights into the neuroendocrine control of inflammation: the role of GR and PARP1. Endocr Connect 3: R1–R12. 24243533 10.1530/EC-13-0079 PMC3869961

[31] Shan L, Li X, Liu L, Ding X, Wang Q, et al.. (2013) GATA3 cooperates with PARP1 to regulate CCND1 transcription through modulating histone H1 incorporation. Oncogene. 10.1038/onc.2013.270 23851505

[32] Maruyama T, Nara K, Yoshikawa H, Suzuki N (2007) Txk, a member of the non-receptor tyrosine kinase of the Tec family, forms a complex with poly(ADP-ribose) polymerase 1 and elongation factor 1alpha and regulates interferon-gamma gene transcription in Th1 cells. Clin Exp Immunol 147: 164–175. 17177976 10.1111/j.1365-2249.2006.03249.x PMC1810450

[33] Sehgal PB (2013) Non-genomic STAT5-dependent effects at the endoplasmic reticulum and Golgi apparatus and STAT6-GFP in mitochondria. JAKSTAT 2: e24860. 24470974 10.4161/jkst.24860 PMC3894245

[34] Sikorski EM, Uo T, Morrison RS, Agarwal A (2006) Pescadillo interacts with the cadmium response element of the human heme oxygenase-1 promoter in renal epithelial cells. J Biol Chem 281: 24423–24430. 16816389 10.1074/jbc.M602287200

[35] Gupta M, Sueblinvong V, Raman J, Jeevanandam V, Gupta MP (2003) Single-stranded DNA-binding proteins PURalpha and PURbeta bind to a purine-rich negative regulatory element of the alpha-myosin heavy chain gene and control transcriptional and translational regulation of the gene expression. Implications in the repression of alpha-myosin heavy chain during heart failure. J Biol Chem 278: 44935–44948. 12933792 10.1074/jbc.M307696200

[36] Kretzschmar M, Kaiser K, Lottspeich F, Meisterernst M (1994) A novel mediator of class II gene transcription with homology to viral immediate-early transcriptional regulators. Cell 78: 525–534. 8062392 10.1016/0092-8674(94)90429-4

[37] Ge H, Roede rRG (1994) Purification, cloning, and characterization of a human coactivator, PC4, that mediates transcriptional activation of class II genes. Cell 78: 513–523. 8062391 10.1016/0092-8674(94)90428-6

[38] Zheng XM, Black D, Chambon P, Egly JM (1990) Sequencing and expression of complementary DNA for the general transcription factor BTF3. Nature 344: 556–559. 2320128 10.1038/344556a0

[39] Keough R, Woollatt E, Crawford J, Sutherland GR, Plummer S, et al (1999) Molecular cloning and chromosomal mapping of the human homologue of MYB binding protein (P160) 1A (MYBBP1A) to 17p13.3. Genomics 62: 483–489. 10644447 10.1006/geno.1999.6035

[40] Verreault A, Kaufman PD, Kobayashi R, Stillman B (1996) Nucleosome assembly by a complex of CAF-1 and acetylated histones H3/H4. Cell 87: 95–104. 8858152 10.1016/s0092-8674(00)81326-4

[41] Zhang Y, Iratni R, Erdjument-Bromage H, Tempst P, Reinberg D (1997) Histone deacetylases and SAP18, a novel polypeptide, are components of a human Sin3 complex. Cell 89: 357–364. 9150135 10.1016/s0092-8674(00)80216-0

[42] Zhang Y, Woodford N, Xia X, Hamburger AW (2003) Repression of E2F1-mediated transcription by the ErbB3 binding protein Ebp1 involves histone deacetylases. Nucleic Acids Res 31: 2168–2177. 12682367 10.1093/nar/gkg318 PMC153746

[43] Guenther MG, Lane WS, Fischle W, Verdin E, Lazar MA, et al (2000) A core SMRT corepressor complex containing HDAC3 and TBL1, a WD40-repeat protein linked to deafness. Genes Dev 14: 1048–1057. 10809664 PMC316569

[44] Li J, Wang J, Wang J, Nawaz Z, Liu JM, et al (2000) Both corepressor proteins SMRT and N-CoR exist in large protein complexes containing HDAC3. EMBO J 19: 4342–4350. 10944117 10.1093/emboj/19.16.4342 PMC302030

[45] Boisvert FM, Chenard CA, Richard S (2005) Protein interfaces in signaling regulated by arginine methylation. Sci STKE 271: re2. 10.1126/stke.2712005re2 15713950

[46] Pal S, Sif S (2007) Interplay between chromatin remodelers and protein arginine methyltransferases. J Cell Physiol 213: 306–315. 17708529 10.1002/jcp.21180

[47] Baranello L, Kouzine F, Levens D (2013) DNA Topoisomerases: Beyond the standard role. Transcription 4. 10.4161/trns.26598 24135702

[48] Nusinzon I, Horvath CM (2003) Interferon-stimulated transcription and innate antiviral immunity require deacetylase activity and histone deacetylase 1. Proc Natl Acad Sci U S A 100: 14742–14747. 14645718 10.1073/pnas.2433987100 PMC299790

[49] Chang H-M, Paulson M, Holko M, Rice CM, Williams BRG, et al (2004) Induction of interferon-stimulated gene expression and antiviral responses require protein deacetylase activity. Proc Natl Acad Sci USA 101: 9578–9583. 15210966 10.1073/pnas.0400567101 PMC470717

[50] Fu Y, Foden JA, Khayter C, Maeder ML, Reyon D, et al (2013) High-frequency off-target mutagenesis induced by CRISPR-Cas nucleases in human cells. Nat Biotechnol 31: 822–826. 23792628 10.1038/nbt.2623 PMC3773023

[51] Hsu PD, Scott DA, Weinstein JA, Ran FA, Konermann S, et al (2013) DNA targeting specificity of RNA-guided Cas9 nucleases. Nat Biotechnol 31: 827–832. 23873081 10.1038/nbt.2647 PMC3969858

[52] Mali P, Aach J, Stranges PB, Esvelt KM, Moosburner M, et al (2013) CAS9 transcriptional activators for target specificity screening and paired nickases for cooperative genome engineering. Nat Biotechnol 31: 833–838. 23907171 10.1038/nbt.2675 PMC3818127

[53] Pattanayak V, Lin S, Guilinger JP, Ma E, Doudna JA, et al (2013) High-throughput profiling of off-target DNA cleavage reveals RNA-programmed Cas9 nuclease specificity. Nat Biotechnol 31: 839–843. 23934178 10.1038/nbt.2673 PMC3782611

[54] Sternberg SH, Redding S, Jinek M, Greene EC, Doudna JA (2013) DNA interrogation by the CRISPR RNA-guided endonuclease Cas9. Nature 507: 62–67. 10.1038/nature13011 PMC4106473 24476820

[55] Rasheed S, Nelson-Rees WA, Toth EM, Arnstein P, Gardner MB (1974) Characterization of a newly derived human sarcoma cell line (HT-1080). Cancer 33: 1027–1033. 4132053 10.1002/1097-0142(197404)33:4<1027::aid-cncr2820330419>3.0.co;2-z

[56] Lozzio CB, Lozzio BB (1975) Human chronic myelogenous leukemia cell-line with positive Philadelphia chromosome. Blood 45: 321–334. 163658

[57] Miller AD, Buttimore C (1986) Redesign of retrovirus packaging cell lines to avoid recombination leading to helper virus production. Mol Cell Biol 6: 2895–2902. 3785217 10.1128/mcb.6.8.2895-2902.1986 PMC367857

[58] Fujita T, Ryser S, Tortola S, Piuz I, Schlegel W (2007) Gene-specific recruitment of positive and negative elongation factors during stimulated transcription of the MKP-1 gene in neuroendocrine cells. Nucleic Acids Res 35: 1007–1017. 17259211 10.1093/nar/gkl1138 PMC1807974

