# Supplementary figures and images for: Identification of Proteins Associated with an IFNγ-Responsive Promoter by a Retroviral Expression System for enChIP Using CRISPR
Source: PLoS One. 2014 Jul 22;9(7):e103084. doi: 10.1371/journal.pone.0103084 (PMC4106880; doi:10.1371/journal.pone.0103084)

Figure S1

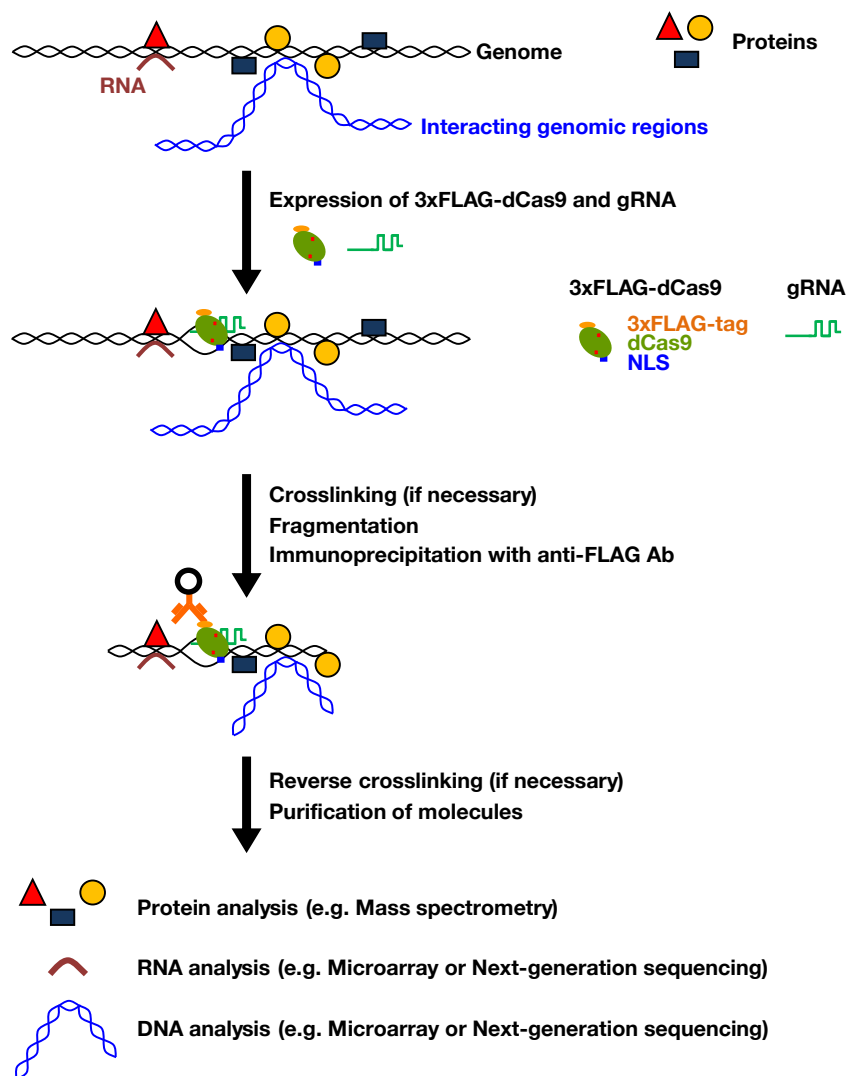

Supplement: Figure S1 — The scheme of enChIP using CRISPR. (PDF) [file pone.0103084.s001.pdf]

**Figure S2**

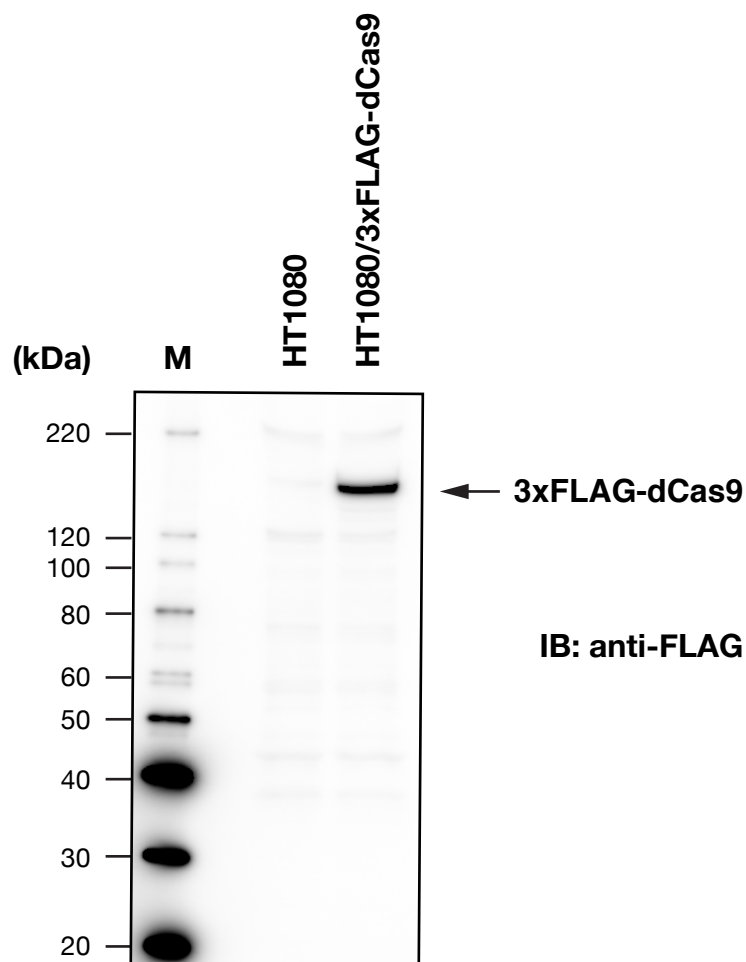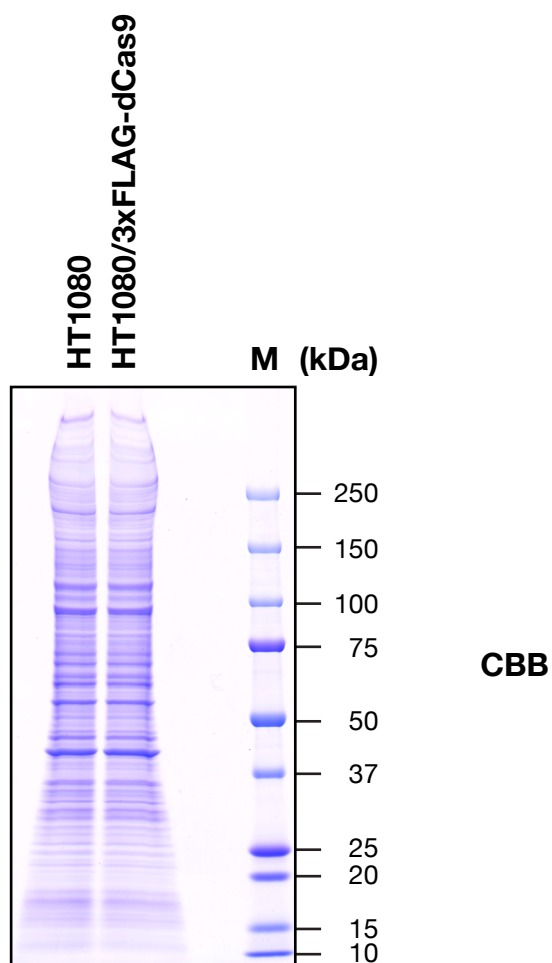

Supplement: Figure S2 — The full-length images of Figure 1A including molecular size markers. (PDF) [file pone.0103084.s002.pdf]

Figure S3

A

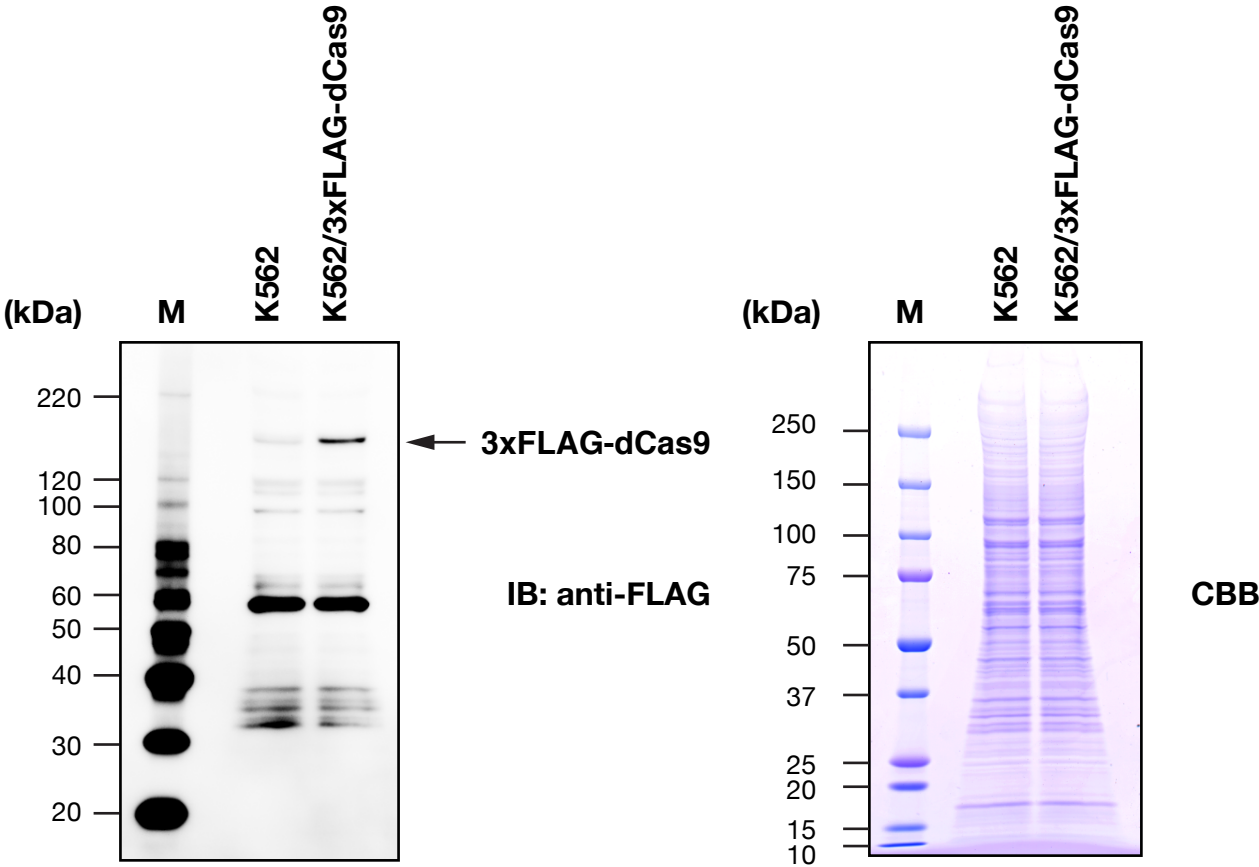

B

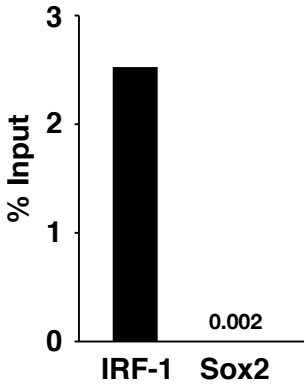

Supplement: Figure S3 — Specific isolation of the IRF-1 promoter region from K562-derived cells by enChIP using CRISPR. (PDF) [file pone.0103084.s003.pdf]

**Figure S4**

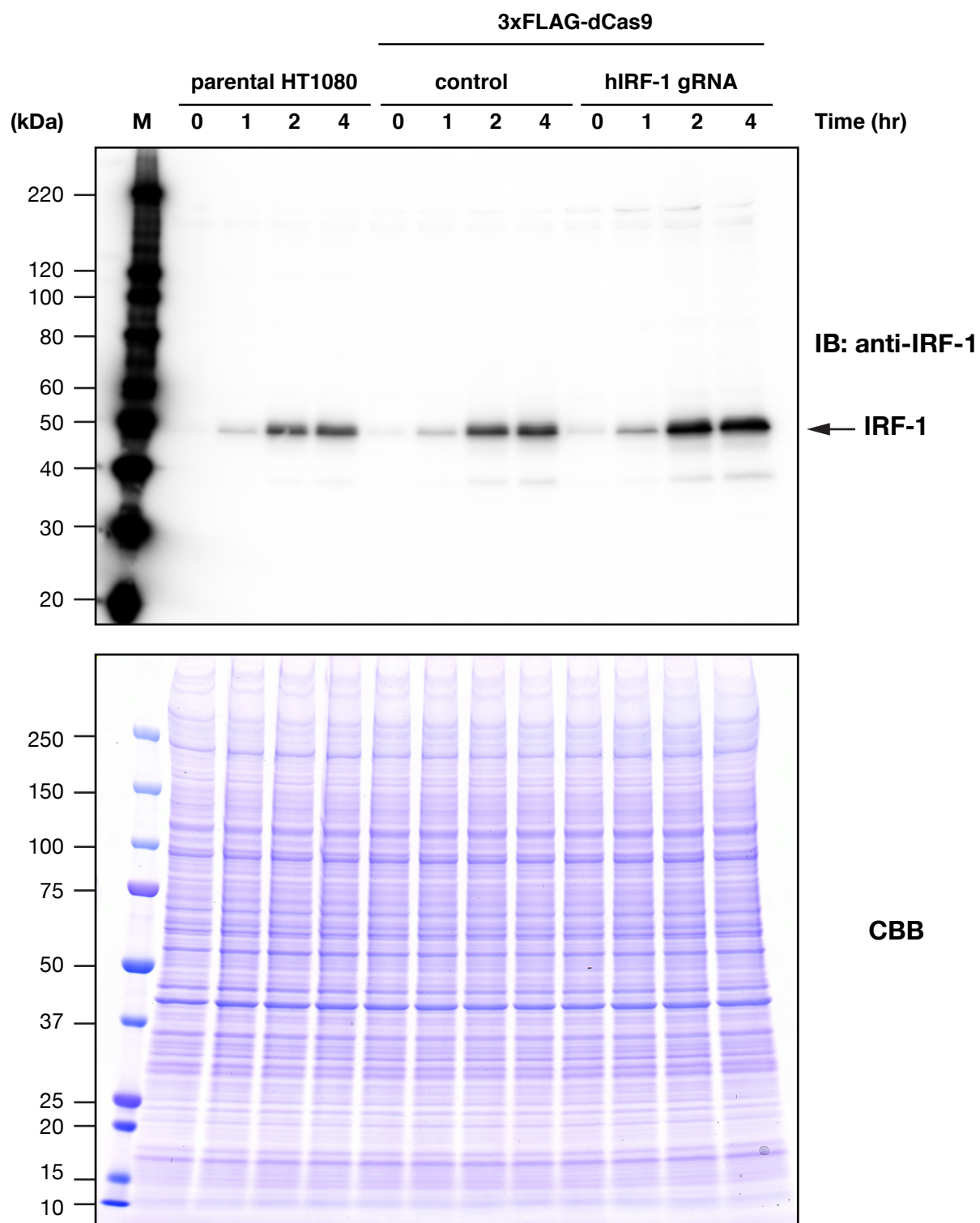

Supplement: Figure S4 — The full-length images of Figure 2 including molecular size markers. (PDF) [file pone.0103084.s004.pdf]

**Figure S5**

**A**

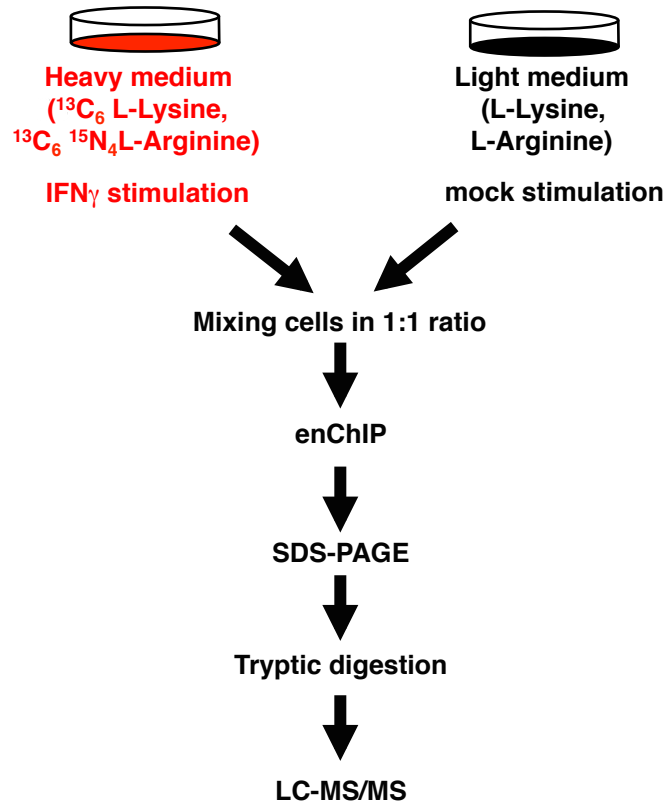

**B**

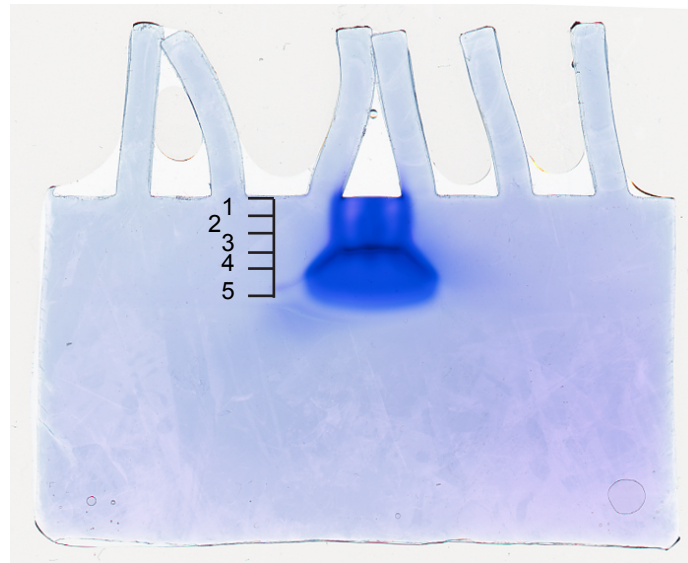

Supplement: Figure S5 — The scheme of enChIP-SILAC. (PDF) [file pone.0103084.s005.pdf]

**Figure S6**

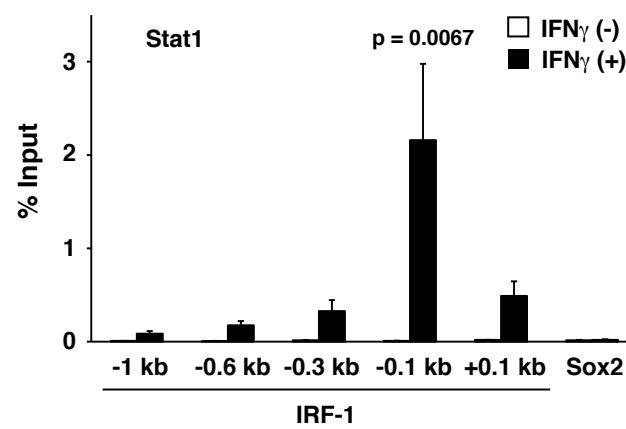

Supplement: Figure S6 — IFNγ-induced association of Stat1 with the IRF-1 promoter. (PDF) [file pone.0103084.s006.pdf]
