# Supplementary material for: Identification of Proteins Associated with an IFNγ-Responsive Promoter by a Retroviral Expression System for enChIP Using CRISPR
Source: PLoS One. 2014 Jul 22;9(7):e103084. doi: 10.1371/journal.pone.0103084 (PMC4106880; doi:10.1371/journal.pone.0103084)
Supplement: Table S2 — Primers used in this study. (PDF) [file pone.0103084.s008.pdf]

Table S2. Primers used in this study

Number	Name	Sequence (5' → 3')	Experiments
27310	hIRF1-prom-F	cgcctgcgttcgggagatatac	enChIP and ChIP assays in Figs. 1 (IRF-1), 3 (-0.3 kb), and S6 (-0.3 kb)
27312	hIRF1-prom-R1+2	ctgtcctctcactccgccttgt	enChIP and ChIP assays in Figs. 1 (IRF-1), 3 (-0.3 kb), and S6 (-0.3 kb)
27222	hSox2-prom-F	attggtcgctagaaacccatttatt	enChIP and ChIP assays in Figs. 1, 3, and S6 (Sox2)
27223	hSox2-prom-R	ctgccttgacaactcctgatacttt	enChIP and ChIP assays in Figs. 1, 3, and S6 (Sox2)
27513	hIRF1-OffTarget-Chr11-F	agcctcagttttcttcttggaca	enChIP assay in Fig. 1 (Chr11)
27514	hIRF1-OffTarget-Chr11-R	ccccaagagtgacaaaagagcta	enChIP assay in Fig. 1 (Chr11)
27507	hIRF1-OffTarget-Chr14-F	cctccctttctgggatttatttg	enChIP assay in Fig. 1 (Chr14)
27508	hIRF1-OffTarget-Chr14-R	tatttggcaggaacactaattgc	enChIP assay in Fig. 1 (Chr14)
27509	hIRF1-OffTarget-Chr17-F	agagcaggaatgacacaaagcag	enChIP assay in Fig. 1 (Chr17)
27510	hIRF1-OffTarget-Chr17-R	gagactacacggaacccagacac	enChIP assay in Fig. 1 (Chr17)
27517	hIRF1-OffTarget-Chr1-F	aaagccacagcgtcctaatctc	enChIP assay in Fig. 1 (Chr1)
27518	hIRF1-OffTarget-Chr1-R	ctccctgacctttgtctccatag	enChIP assay in Fig. 1 (Chr1)
27519	hIRF1-OffTarget-Chr22-F	cgagggcctagtcgtgtcct	enChIP assay in Fig. 1 (Chr22)
27520	hIRF1-OffTarget-Chr22-R	cagctgtctgcacccacagt	enChIP assay in Fig. 1 (Chr22)
27561	hIRF1-(-1k)-F	gggactcagagatgacagcaatg	ChIP assay in Figs. 3 and S6 (-1 kb)
27562	hIRF1-(-1k)-R	tctgatccctcctagcactcttg	ChIP assay in Figs. 3 and S6 (-1 kb)
27416	hIRF1-prom-F5	gtaggctttctgcctccttcact	ChIP assay in Figs. 3 and S6 (-0.6 kb)
27417	hIRF1-prom-R5	atcaagaagggagggtttcagtc	ChIP assay in Figs. 3 and S6 (-0.6 kb)
27567	hIRF1-(-0.1k)-F	agctctacaacagcctgatttcc	ChIP assay in Figs. 3 and S6 (-0.1 kb)
27568	hIRF1-(-0.1k)-R	aatccaaacacttagcgggattc	ChIP assay in Figs. 3 and S6 (-0.1 kb)
27563	hIRF1-(+0.1k)-F	actccttagtcgaggcaagacg	ChIP assay in Figs. 3 and S6 (+0.1 kb)
27564	hIRF1-(+0.1k)-R	aagagggaagaaggcagaggtt	ChIP assay in Figs. 3 and S6 (+0.1 kb)
